# Character and Uses of Saliva.—Correction

**Published:** 1868-11

**Authors:** Henry S. Chase

**Affiliations:** 216 North Sixth St. Saint Louis.


### CORRESPONDENCE.

---

### ARTICLE VI.

### *Character and Uses of Saliva.—Correction.*

Mr. Editor :—As I see that the Reports of the American Dental Association, in both the *Am. Journal* and *Cosmos* are incorrect, so far as it relates to my paper on the "Character and Uses of Saliva." I wish to say a few words in relation to the subject.

In that paper I stated that I had examined a large number of *healthy* animals and found the saliva invariably *alkaline.* The *Reporter* makes me say acid. I also stated that

I had examined a large nnmber of unhealthy animals, or those at least living in an *artificial* state, unfavorable to health, and found *their* saliva to be either acid or neutral. In a large number of men women and children I fouud the saliva to be *acid*, excepting at meal time, or a few moments after eating.

The *Reporter* says I " concluded that healthy saliva is not neutral in its reaction in man or in the lower animals" My paper states my conclusions to be that both in man and in the lower animals, living in a *natural* condition,—perfect physiological condition—that the " mixed saliva of the mouth " *is* either *neutral* or *alkaline.*

To sum up in fewer words:

Man or the lower animals being in

The Perfect Physiological State—Alkaline Saliva.
Partially " " —Neutral.
A less " " —Acid.

During the mastication of food the saliva approaches more or less perfectly the *alkaline* condition, depending on the degree of health possessed by the individual.

HENRY S. CHASE,
216 North Sixth St. Saint Louis.

---

